# Aryl Guanyl Hydrazones: A Viable Strategy for Designing BBB-Permeable, Neuroactive Compounds?

**DOI:** 10.1021/acschemneuro.5c00463

**Published:** 2025-07-22

**Authors:** Eleonora Colombo, Leonardo Maiorana, Greta Donati, Andrea Menegon, Nicoletta Collura, Luca Muzio, Eloise Mastrangelo, Mario Milani, Luciana Marinelli, Pierfausto Seneci

**Affiliations:** † Chemistry Department, 9304 Università degli Studi di Milano, Via Golgi 19, 20133 Milan, Italy; ‡ Neuroscience Division, San Raffaele Scientific Institute, Via Olgettina 58, 20132 Milan, Italy; § Department of Pharmacy, Università degli Studi di Napoli Federico II, via D. Montesano 49, 80131 Napoli, Italy; ∥ Experimental Imaging Centre, San Raffaele Scientific Institute, Via Olgettina 58, 20132 Milan, Italy; ⊥ Institute of Biophysics (IBF), CNR, Via Celoria 26, 20133 Milan, Italy

**Keywords:** aryl guanyl hydrazones, tautomeric equilibria, BBB, p*K*_a_, rational drug design, NMR, nucleic acid binding, bioavailability

## Abstract

Guanyl hydrazones are emerging as valuable, target-tunable functional groups. In particular, aryl guanyl hydrazones, owing to extensive conjugation with aromatic rings, exhibit lower p*K*_a_ values compared to their aliphatic counterparts, so that, at physiological pH, a substantial proportion of them remains in a nonionized or partially protonated form, thereby increasing their lipophilicity and enhancing BBB permeability. Intriguingly, their tautomeric equilibria provide flexible charge allocation, adapting to binding site demands for protein and nucleic acid target species. Herein, (hetero)­aromatic drugs, clinical candidates, leads and hits bearing one or more guanyl hydrazones are presented in terms of mechanism of action, *in vitro* and *in vivo* potency. Synthetic access to guanyl hydrazone-containing molecules, through complementary and simple routes, is briefly presented. Future trends for aryl guanyl hydrazones in CNS and PNS drug discovery are critically discussed.

## Graphical abstract

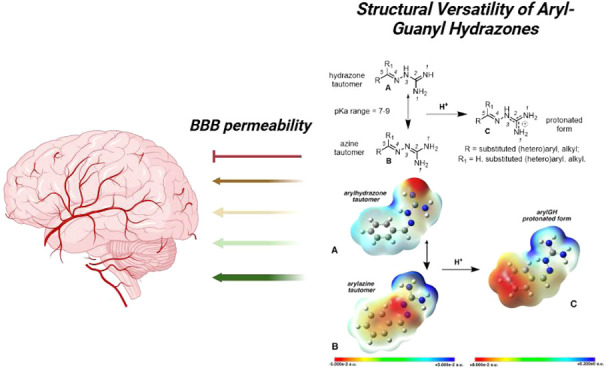

## 1 BBB-Compliant Small Organic Molecules

Amphiphilic biological membranes^1^ are selectively permeable barriers that limit the access of molecules to cells (cell membranes) or to cellular compartments (intracellular membranes), depending either on size and charge (passive diffusion), or on energy-dependent mechanisms (active transport, against a concentration gradient). Tissue barriers built on junctional complexes^2^ regulate the access to human tissues, preserve their functionality, and their dysfunctional status is observed in multiple diseases.^3^

The blood-brain barrier (BBB^4^), and its blood-nerve (BNB^5^) and blood-spinal cord (BSCB^6^) complements, tightly regulate the access of molecules, ions and cells to the central and peripheral nervous system (CNS and PNS, respectively),^7^ protecting from inflammation, toxins, pathogens and other disease-causing agents.^4^ Dysfunctional CNS and PNS barriers result from neurologic diseases, and may even be their causative factor.^4,7^ To treat such diseases, symptomatic and disease-modifying treatments must access their molecular targets by crossing the BBB.

Small lipophilic molecules passively diffuse through the highly lipophilic BBB;^8^ conversely, charged molecules at physiological pH are likely excluded from the brain, or peripheral nerves.^9,10^ In this Review we describe aryl guanyl hydrazones that, owing to conjugation with (hetero)­aromatic rings, exhibit lower p*K*_a_ values compared to their aliphatic counterparts, so that, at physiological pH, a substantial proportion of these compounds remains in a nonionized or less protonated form, thereby increasing their lipophilicity and enhancing BBB permeability.

## 2 Guanyl Hydrazones: Molecular Properties

Guanyl hydrazones (GHs from now on) are moderately basic groups at physiological pH, with p*K*_a_ values typically ranging between 6.5 and 9.^11,12^ They consist of a substituted 5-atom chain with four nitrogen atoms, one N–N, two C–N and two C=N bonds. The latter bonds arrange either in a 1,4 nonconjugated or in a 2,4- conjugated tautomeric form (respectively hydrazone **A** and azine **B**, Figure 1), coalescing in a single protonated form **C** upon acidification (delocalized charge between the two N_1_ and the N_3_ atoms).

**Figure 1:**
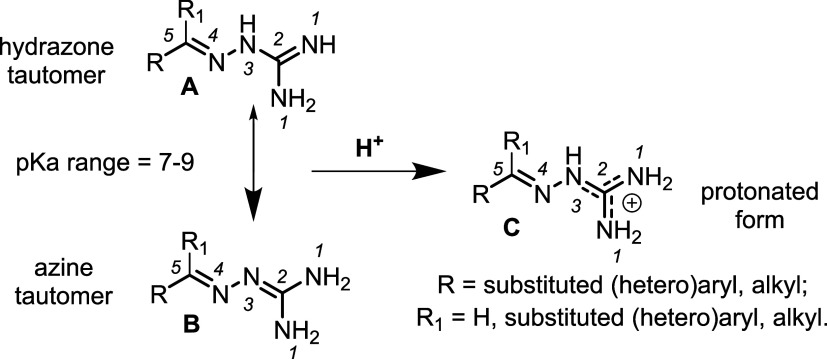
GHs: hydrazone – azine (A–B) tautomeric equilibrium, single protonated form (C).

Quantum chemical calculations^13,14^ and experimental evidence (IR - LC/MS - NMR,^13^ crystallography^15,16^ ) indicate a 4–6 kcal/mol stabilization preference for the azine vs the hydrazone tautomer; bis-GHs show up to 12 kcal/mol tautomer stabilization.^17^ A slow azine-hydrazone equilibrium is suggested by Nuclear Overhauser (NOE) NMR experiments.^15^

Polar, tautomeric GH groups are promising substitutions for biologically active compounds. Their charge delocalization on multiple N atoms elicits strong ionic interactions in suitably shaped binding sites; the different electronic map of azine vs hydrazone tautomers^14^ suggests that GHs are able to switch between isoforms in a target-induced mode and with a relatively low energy penalty, depending on target binding requirements.

The molecular electrostatic potential maps (MEPs)^18^ of aryl GHs highlight a nonhomogeneous electron density distribution among noncharged tautomers (Figure 2).

**Figure 2:**
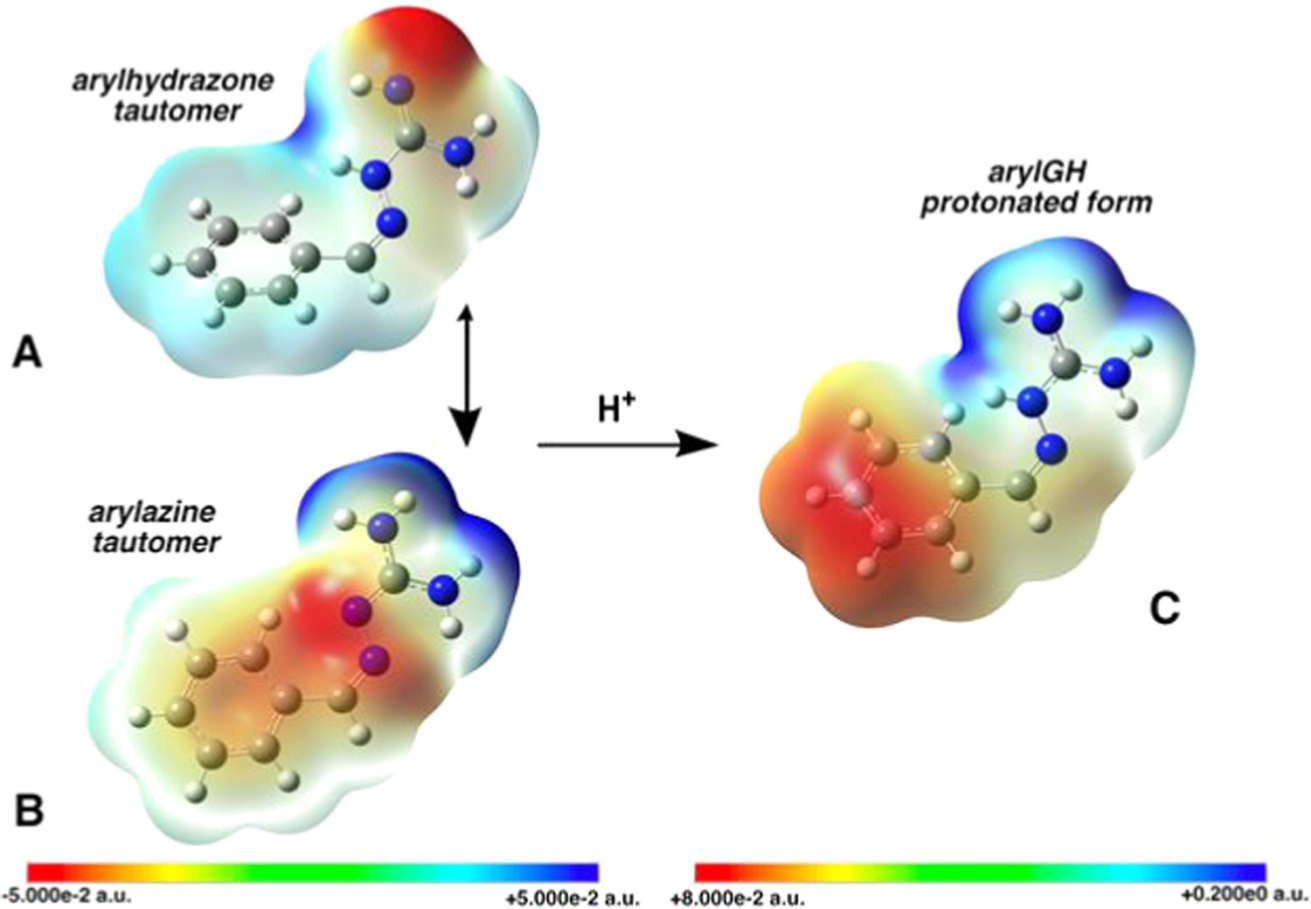
Free hydrazone (A) vs free azine (B) tautomers, and protonated form (C) of aryl GHs:.

In the disfavored arylhydrazone tautomer (Figure 2A), an N_1_ atom (see Figure 1 for numbering) is bound to an H atom and is characterized by a larger electron density (red end of the palette), while the other N_1_ and the N_3_ atom are electron-poor. In addition, the two nonconjugated C=N bonds cause an asymmetrical spread of the electron density (Figure 2A).

The favored arylazine GH tautomer shows an electron-poor region spread across both N_1_ atoms, and a higher electron density on the N_3_–N_4_ portion (Figure 2B). Its C=N bonds are fully conjugated with the aryl moiety, coherently with lower measured p*K*_a_ values and with an almost homogeneous electronic density distribution spanning the region from the aromatic ring to the N_3_ atom.

In the protonated aryl GH form the positive charge is homogeneously spread along the two N_1_ atoms and the N_3_ atom (Figure 2C), and partial aryl ring conjugation is preserved.

Additionally, GHs may experience an E/Z equilibrium at their C_5_ position. If R_1_ is H, the E form is preferred; the same is true for 5-disubstituted GHs (estimated ΔGs between 0.5 and 6.5 kcal/mol^13^), with rare exceptions involving similar R and R_1_ substitutions.

## 3 Synthetic Access to Guanyl Hydrazones

GHs are easily accessible through a condensation between aminoguanidine (AG from now on) salts and carbonyl compounds (aldehydes, R_1_=H; ketones, R,R_1_≠H, Scheme 1).

**Scheme 1:**
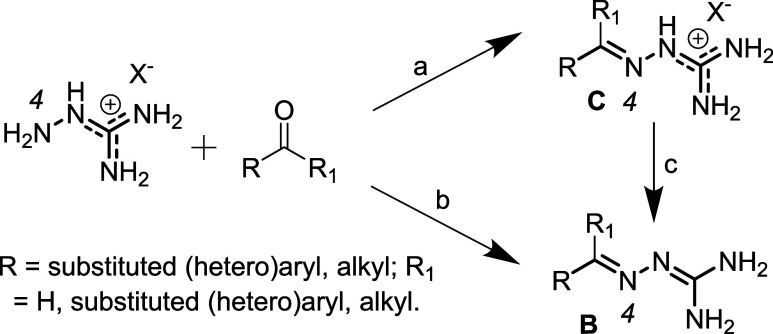
Synthetic Routes to GH Salts (a) and Free Bases (b, c)^a^

a a: cat. H^+^, refluxing EtOH, up to 8 h; b: 1N NaOH (2 equiv), H_2_O, rt, up to 1 h; c: 1N NaOH, MeOH, 50°C, 4 h.

An acid-catalyzed condensation (catalytic H^+^, a, Scheme 1) in refluxing EtOH should be carried out at pH ≥ 2, to ensure the presence of a free, nucleophilic N_4_ atom and of a delocalized positive charge on other N atoms, to limit side reactions;^19^ experimental protocols entail AG.HCl,^20^ AG.H_2_SO_4_ or AG.HNO_3_.^21^ Milder experimental conditions, shorter reaction times and higher yields may be achieved by ultrasound-assisted^22^ and microwave-assisted synthesis.^23^

AG.HCl reacts with a carbonyl compound in water, in the presence of excess NaOH (basic, b, Scheme 1); higher yields due to precipitation of pure GHs as free bases, shorter reaction times and lower temperatures are claimed.^13^ Free GHs may be obtained by neutralizing their salts in stronger basic conditions (c, Scheme 1).^13,15^

GH salts often crystallize while cooling the reaction mixture, and can be stored indefinitely; hydrochlorides are used for *in vitro*/cellular biological testing, while GH salts with organic acids are often used to increase bioavailability *in vivo*.

Many commercial/easily prepared substituted carbonyl compounds, easy synthetic access to N-substituted AGs,^24^ and the compatibility of AG condensation protocols with most functional groups are the gateway to multiple GH-bearing, biologically active compound classes, and to the acquisition of detailed Structure–Activity Relationships (SARs) around them. Scaffold assembly and functionalization occur first, with GHs being introduced as a last synthetic step due to their poor solubility in organic solvents and simple purification protocols.

GHs were synthesized, structurally optimized and clinically tested against systemic indications (Figure 3). Free AG, named **pimagedine**,^25^ was tested against diabetic nephropathy in >1000 patients; it missed efficacy end points and caused side effects.^26^ The alkyl bis-GH **mitoguazone** (MGBG) was clinically tested in multiple trials,^27^ showed suboptimal efficacy and toxicity on AIDS-associated non Hodgkin lymphoma (NHL), and eventually was abandoned.^28^ The amidinoindane GH **sardomozide** (SAM486A, CGP-48664)^29^ was clinically tested against refractory NHL^30^ and metastatic melanoma,^31^ resulting to be safe but inactive.

**Figure 3:**
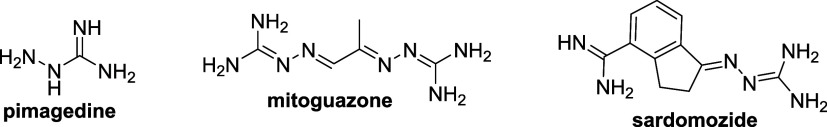
GH-containing clinical candidates against systemic indications.

## 4 Aryl Guanyl Hydrazones as CNS-Active Drugs, Candidates, Leads and Hits

The moderate p*K*_a_ of aliphatic GHs is lowered by extended conjugation in (hetero)­aryl GHs. In fact, therapeutically relevant brain levels of several CNS-active aryl GH drugs or candidates, shown in Figure 4, have been bioanalytically certified.

**Figure 4:**
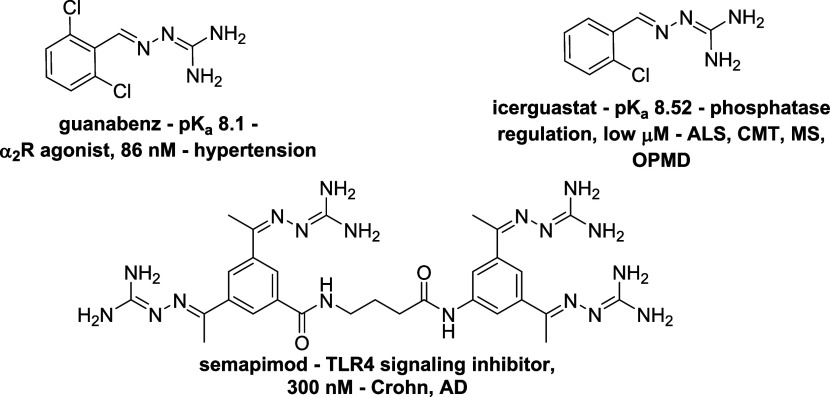
BBB-permeable aryl GH drugs and candidates.

The nanomolar antihypertensive α_2_-adrenergic receptor agonist dichlorophenyl GH drug **guanabenz** has a 8.1 p*K*_a_ value, corresponding to a ≈16% free base at 7.4 pH;^32^ its p*K*_a_ is lowered by extensive conjugation, and by two electron-withdrawing chlorine atoms.^21^ Its orally bioavailable acetate salt^33^ showed a pharmacokinetic (PK) profile after iv injection^34^ entailing ≈4 ng/mL plasma levels after 5 min at the lowest 32 μg/kg effective dosage, followed by rapid plasma disappearance; and maximal ≈30 ng/mL brain levels after 15 min, followed by a slow elimination rate (≈15 ng/mL, 3 h postinjection).

Chlorophenyl GH **icerguastat** (IFN-088, sephin1) shows a slightly higher p*K*_a_ than guanabenz, due to the removal of a chlorine atom.^35^ Its orally administered acetate salt (10 mg/kg, rats) led to ≈20 ng/mL plasma levels after 30 min, rapidly declining to <10 ng/mL; brain and PNS/sciatic nerve levels peak respectively to ≈200 and ≈170 ng/mL after 30 min, then being slowly eliminated (respectively ≈35 and ≈50 ng/mL after 8 h).^36^ Icerguastat acts by inhibiting the regulatory protein phosphatase 1 (PP1),^36^ and/or by inhibiting holophosphatase 2a (PP2A) assembly;^37^ it has successfully completed a Phase II trial against bulbar-onset Amyotrophic Lateral Sclerosis (ALS) (NCT05508074 ^38^), and has shown *in vivo* efficacy in mice models of Charcot-Marie Tooth (CMT) disease and ALS,^36^ Multiple Sclerosis (MS)^39^ and OculoPharyngeal Muscular Dystrophy (OPMD).^40^

Diaryl tetra GH **semapimod**(CNI-1493) is a nanomolar suppressor of pro-inflammatory cytokine production through inhibition of Toll-like receptor 4 (TLR4) signaling;^41^ notwithstanding its higher p*K*_a_ value due to four GHs, it showed ≈2 ng/mL brain levels 1 h after iv injection of its ^14^C-radiolabeled free base (1 mg/kg).^42^ Its tetrahydrochloride salt was clinically tested against Crohn disease,^43^ and showed *in vivo* activity against multiple indications;^44^ an orally bioavailable salt formulation (CSPI-2364) was also reported.^45^ Semapimod showed biochemical evidence (Aβ clearance) and behavioral end points (preservation of object recognition memory) as a neuroprotective microglial activation-inhibiting agent after i.p. administration in a TgCRND8 Alzheimer’s disease (AD) mouse model;^46^ and prevented paralysis after early therapeutic i.p. administration in an experimental autoimmune encephalomyelitis (EAE) mouse model of MS.^47^

Such examples do not grant BBB permeability to each and every aryl GH, but support the consideration for suitably functionalized aryl GH as BBB-compliant small molecules. This is further supported by the recognition of particular GHs by sodium ion channels, leading to BBB permeation.^48^ Multiple **e**xamples of neuroactive aryl GH hits and leads are shown in Figure 5, and are briefly presented here.

**Figure 5:**
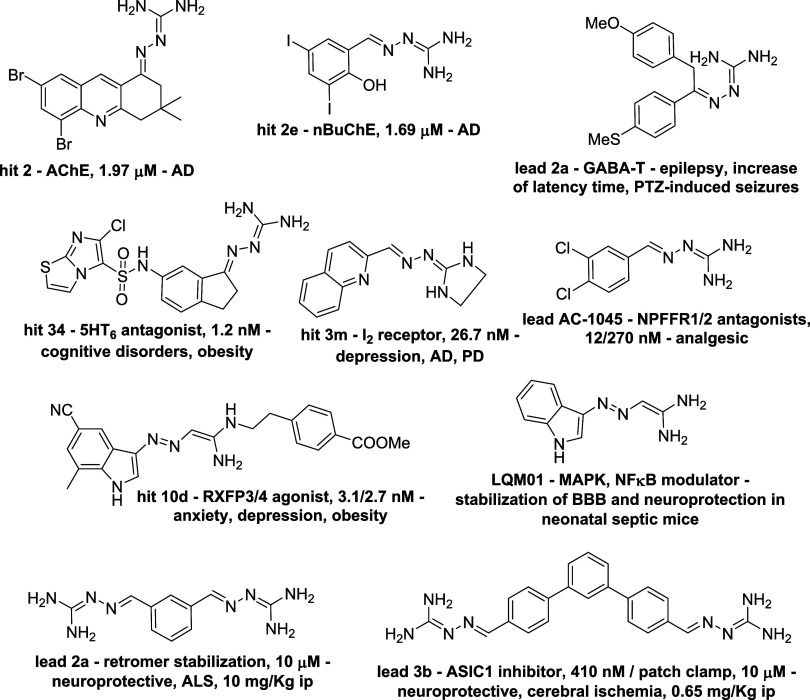
Neuroprotective, BBB/BNB/BSCB-compliant GH hits and leads.

GH leads and hits inhibiting neuronal enzyme targets include tricyclic dibromo GH hit **2**,^49^ a potent acetylcholinesterase (AChE) inhibitor and putative symptomatic AD treatment, whose molecular interactions within the AChE binding site were elucidated through modeling studies. Trisubstituted diiodophenol GH hit **2e** ^50^ shows good selectivity for closely related butyrylcholinesterase (BuChE) vs AChE enzymes, and lacks toxicity in hepatic cell lines. 1,2-Diarylethylidene, 5-disubstituted GH lead **2a** ^51^ shows *in vivo* anticonvulsant activity, possibly through inhibition of γ-aminobutyric acid (GABA) transaminase, and protects rats from (PTZ)-induced seizures in an epilepsy model; its (E)-configuration was analytically confirmed.

As to neuronal receptor targets, sulfonamidoaryl GH hit **34** ^52^ is a low nanomolar, full antagonist of the 5-hydroxytriptamine 6 (5-HT_6_) receptor, with good selectivity vs other serotoninergic and adrenergic receptors, as a putative treatment against dementia and obesity. N_1_-cyclized quinoline GH hit **3m** ^53^ is a nanomolar, selective imidazoline 2 (I_2_) receptor antagonist, relevant against depression and neurodegenerative diseases. Dichlorophenyl GH lead **AC-1045** ^54^ is a potent neuropeptide FF receptor (NPFF) antagonist; its debatable aspecificity on the NPFF-1 and −2 receptor isoforms^55^ may contribute to its analgesic profile in rodents. N_1_–Substituted indole GH lead **10d** ^56^ is a BBB-permeable, low nanomolar agonist of the relaxin family peptide receptors 3 and 4 (RXFP3/4), with anxiolytic and antidepressant potential.*In vivo*-neuroprotective, mitogen-activated protein kinase (MAPK) and nuclear factor kappa-light-chain-enhancer of activated B cells (NF-κB) modulator indole GH lead **LMQ01** shows a neuroprotective BBB stabilization after lipopolysaccharide (LPS)-caused neuroinflammation and septic shock in neonatal mice.^57^

Our group reported two BBB-permeable, *in vivo* active GH leads. *Meta* triphenyl *para* bis-GH **3b**(ref 20, Figure 5) is a human ASIC1a nanomolar inhibitor with partial selectivity vs ASIC2, whose patch clamp electrophysiology profile at 10 μM (Figure 6) shows a substantial inhibition of currents elicited by pH 6.5 in cells expressing human ASIC1a. Its effectiveness in a middle cerebral arteria occlusion (MCAO) mouse model, and an established preliminary SAR bode well for the future development of innovative aryl GH treatments against cerebral ischemia.

**Figure 6:**
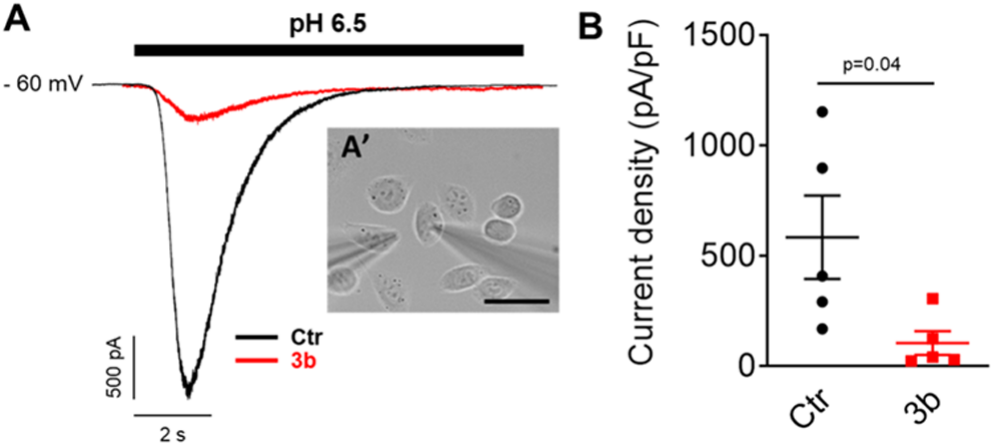
(A) Inhibition of inward currents elicited by pH 6.5 stimulation in CHO-K1 cells expressing Human ASIC1a (hASIC1a-CHO-k1): pH evoked current alone (black), and after treatment with **3b** (10 μM, red); **A’**, phase contrast image of a hASIC1a-CHO-K1 cell with a patch pipet (right) and a puff pipet placed near the cell (left). (B) Quantification of peak current amplitudes induced in control conditions (black dots), and after application of **3b** (red squares, 10 μM). Data show the mean ± SEM (*n* = 5 for each group). Statistic: t Test, unpaired. Scale bar= 20 μm.

*Meta* phenyl bis-GH **2a** ^58^ is a pharmacological chaperone binding at the interface between VPS29 and VPS35, two key protein components of the cargo recognition core (CRC) of the retromer complex; its BBB permeability and CNS levels at effective concentrations were determined through a preliminary, 7 days’ quantification in the brain of naïve C57LB6 mice (Figure 7), showing the accumulation of **2a** in their brains after daily ip injection up to therapeutically significant 6 ng/mg levels in brain tissue. The cellular potency of **2a** as a retromer stabilizer, its *in vivo* efficacy in a SOD1 ALS mouse model, and a detailed understanding of its molecular interactions with the retromer^59^ grant further optimization toward disease-modifier drugs against retromer-influenced neurodegenerative diseases.

**Figure 7:**
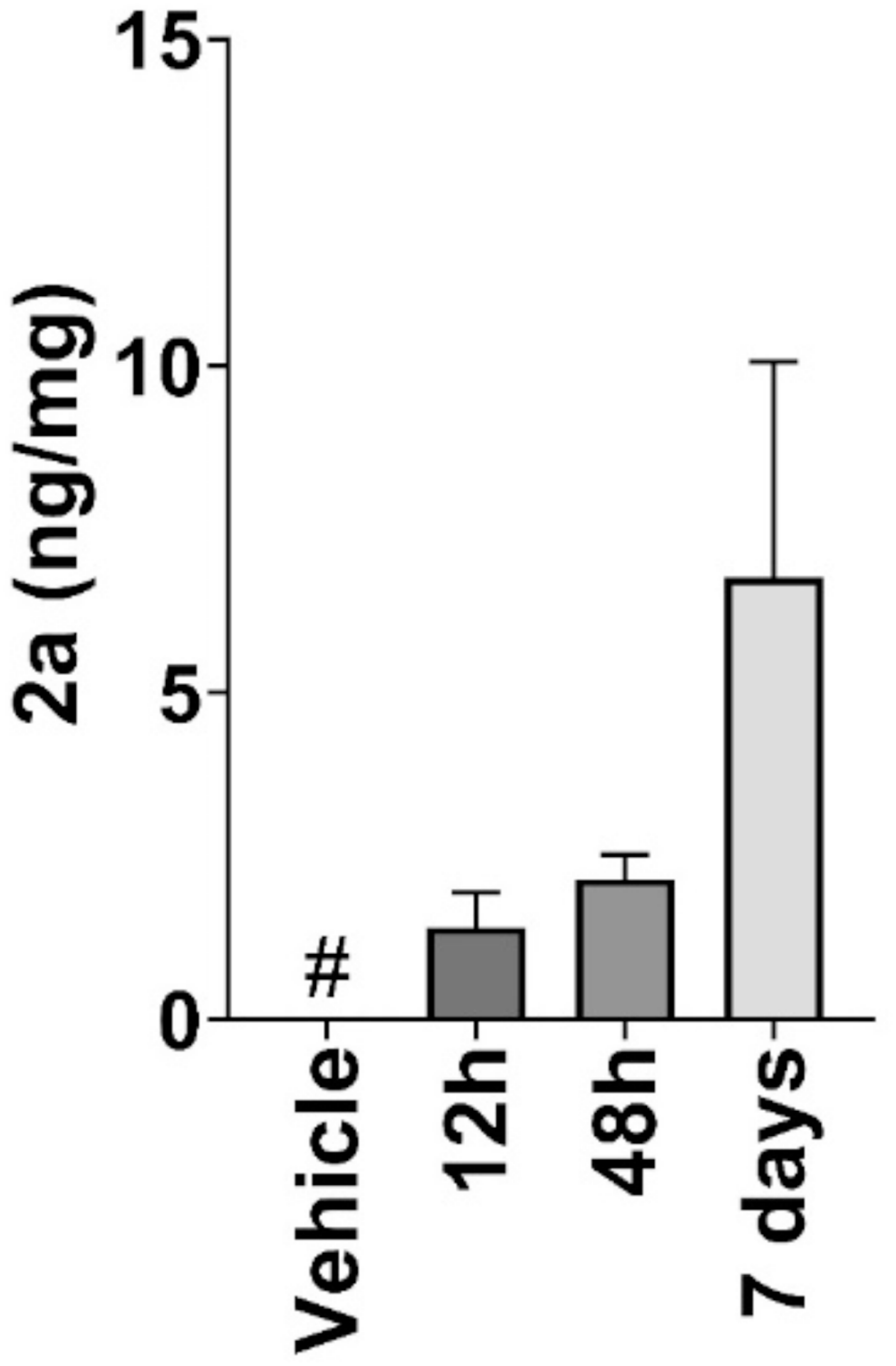
Time-course of brain uptake in naive C57BL6 mice injected daily with 10 mg/kg of **2a** (*n* = 3 for each group). CNS explants were dissected from mice receiving **2a** injections, and their **2a** levels were determined by mass spectrometry. Data show the mean ± sd (*n* = 3 for each group). One-way ANOVA followed by Tukey’ multiple comparisons tests.

## 5 Aryl Guanyl Hydrazones as Nucleic Acid Binders

Small molecules interacting with either DNA (i.e., intercalating agents^60^) and RNA (i.e., streptomycin^61^) are well-known since many decades. Recently, specific binders to nucleic acid secondary structures, such as G-quadruplexes (G4s)^62^ and stem loops/hairpins,^63^ have been characterized, taking advantage of computational DNA and RNA models and biophysical studies.^64^ RNA targeting either with small molecule RNA binders or through modulation of RNA-binding proteins (RBPs^65^) has gained attention, due to the broad influence of RNA species (i.e., messenger/mRNAs, long noncoding/lncRNAs, micro- and small interference/miRNAs and siRNAs) on pathophysiological processes.^66^

A set of small molecule modulators has been developed to selectively target diverse secondary DNA and RNA structures.^67^ Privileged scaffolds are well established for protein–ligand interactions,^68^ but were recently identified also for nucleic acids. Namely, DNA- or RNA-targeted, G4- or hairpin-specific small molecules show polyaromatic, delocalized π-systems, and one or two positively charged substituents at physiological pH – the latter to exploit binding to single- and double stranded nucleotide targets.

Figure 8 shows three hits built on such privileged, RNA-interacting structures. Benzamide-centered diamidinoamine **2**, binding to expanded CUG hairpin RNA repeats against myotonic dystrophy;^69^ indole-centered diamidine **synucleozid**, targeting the iron responsive element (IRE) hairpin in α-synuclein mRNA^70^ as a putative PD lead; and symmetrical diphenylthiophene diamidine **DB1247**, targeting expanded G_4_C_2_ G4 RNA repeats in c9ORF72 against ALS.^71^

**Figure 8:**
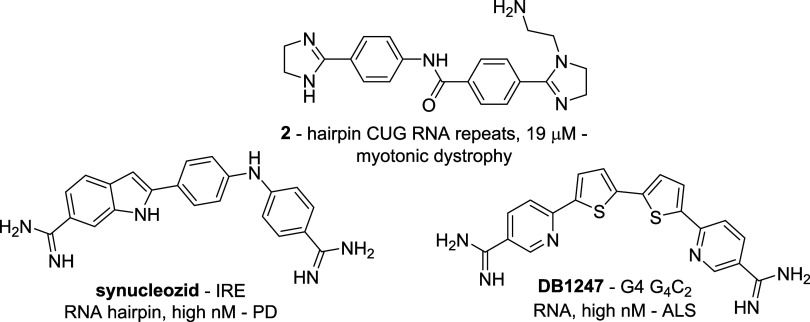
Basic polyaromatic DNA and RNA binders.

Biophysical, cell-free and cellular assay results were reported for such diamidines; the need for bioavailable analogues suitable for in *vivo s*tudies was often mentioned, due to their poor permeability through membranes caused by physiologically ionized diamidines.

Mono- and bis-aryl GHs DNA and RNA binders, possibly endowed with better bioavailability due to a lower p*K*_a_, are shown in Figure 9. Systemic agents include antifungal phenylfuran bis-GH hit **BG3**,^72^ and 1,10-phenantroline-centered anticancer bis-GH hit **PhenQE8**,^73^ diimidazopyrimidine mono-GH hit **3** ^74^ and phenol-centered mono-GH hit **15**.^75^

**Figure 9:**
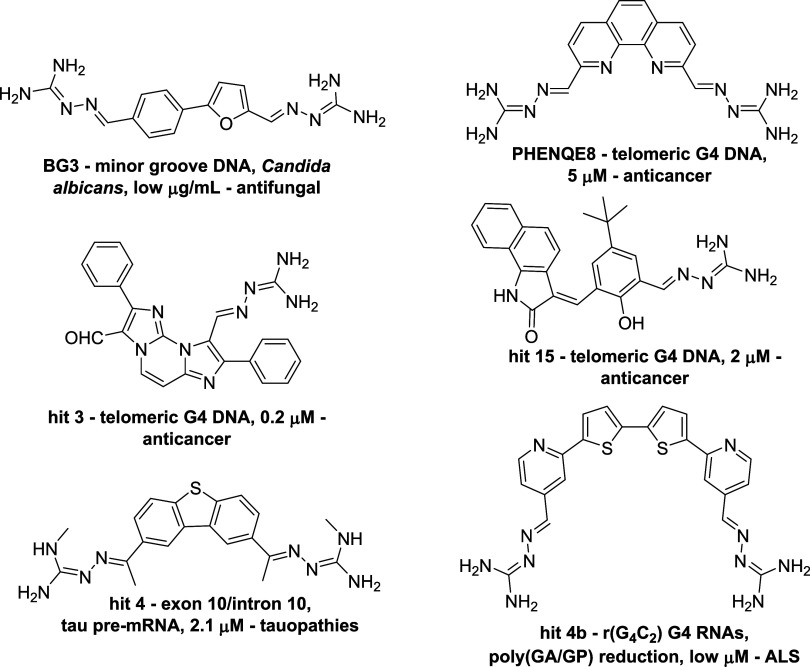
Polyaromatic mono- and bis GHs as DNA and RNA binders.

As to BBB-compliant agents, dibenzothiophene bis-GH hit **4** interacts with a bulge region in the pre-mRNA sequence of tau, acting as a pre mRNA splicing modifier^76^ and rebalancing the 3*R*/4R tau isoform ratio, typical in tauopathies, by exclusion of exon 10 at low micromolar concentrations. We reported diphenylthiophene di-GH hit **4b** ^77^ as a bioavailable structural analogue of earlier mentioned diamidine DB1247 (Figure 8), binding to expanded repeats in ALS-determining c9ORF72 and preventing poly­(GA/GP) translation at low micromolar concentrations. The preferential binding of **4b** to G4-structured nucleic acids was experimentally confirmed by NMR spectra, as shown in Figure 10, where the significant spectral changes in the absence (blue track) and presence (black track) of **4b** indicate a nucleic acid-**4b** interaction.

**Figure 10:**
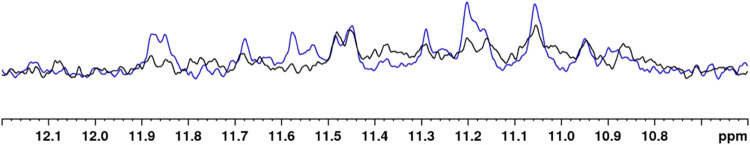
1D ^1^H NMR spectrum (imino proton region) of the G4 DNA sequence c-kit2 at 15 μM (blue spectrum), recorded in buffer containing 100 mM KCl and 10 mM Tris at pH 7.0, before and after the addition of **4b** at 60 μM (black spectrum).

By replacing amidine, guanidine or amine groups with GHs in DNA- and RNA-binding small molecules, bioavailability and flexibility should be increased to address their target sequence, once the delocalized π-system has been tailored to fit one or two appropriately placed GHs. Leveraging biophysical and modeling studies on RNA species associated with neurological conditions, could further expand the current panel of poly aromatic scaffolds and GH-bearing substituents, and could enable tailoring the resulting aryl mono- and bis GHs to diverse therapeutic needs.

## 6 Conclusions

Biologically active aryl GH hits, leads and drug candidates have progressively gained attention in the scientific literature over the past decades. Their synthetic accessibility and versatility, metabolic stability and bioavailability, combined with their favorable molecular interactions within selected target binding sites (including both proteins and nucleic acids), suggest a growing role for GHs as valuable moieties in drug discovery. We foresee an increasing number of GH moieties in the rational design of biologically active compounds in general, and particularly in the development of neuroactive agents.

A better understanding of the impact of structural modifications of aryl GH hits both on the aryl portion (including polycycles and heteroaryl groups), and on the poorly exploited GH structure (including mono-, polyalkylated and cyclic GHs) is essential to further expand their usefulness. Exploring these modifications could enhance their key physicochemical properties and bioavailability, and optimize their drug-likeness, particularly in relation to CNS compliance. Given their potential as versatile scaffolds for high-throughput analogue design and optimization, aryl GHs are poised to become increasingly relevant in medicinal chemistry, offering promising avenues for the discovery of novel therapeutic agents.

## Abbreviations

AD: Alzheimer’s disease
AG: aminoguanidine
ALS: amyotrophic lateral sclerosis
BBB: blood brain barrier
BNB: blood nerve barrier
CNS: central nervous system
E/Z: *entgegen/zusammen*
GHs: guanyl hydrazones
HD: Huntington’s disease
IR: infrared
LC: liquid chromatography
MEP: molecular electrostatic potential
MS: mass spectrometry
NOE: nuclear Overhauser effect
NMR: nuclear magnetic resonance
PD: Parkinson’s disease
PNS: peripheral nervous system
SARs: structure–activity relationships
vHTS: virtual high throughput screening.
